# Predicting Proximal Femoral Remodeling After Short-Stem Hip Arthroplasty: A Biomechanical Modeling Approach

**DOI:** 10.3390/jcm14155307

**Published:** 2025-07-27

**Authors:** Jan Heřt, Martin Havránek, Matej Daniel, Antonín Sosna

**Affiliations:** 1 1st Department of Orthopaedics, Motol University Hospital, 150 06 Prague, Czech Republic; 2 Department of Mechanics, Biomechanics and Mechatronics, Czech Technical University in Prague, 166 36 Prague, Czech Republic; matej.daniel@fs.cvut.cz

**Keywords:** short stem, total hip arthroplasty, biomechanical modeling

## Abstract

**Background**: Short-stem hip replacements are designed to provide improved load distribution and to mimic natural biomechanics. The interplay between implant design, positioning, and resulting bone biomechanics in individual patients remains underexplored, and the relationship between radiographically assessed bone remodeling around short stems and biomechanical predictions has not been previously reported. **Methods**: This study evaluated three short-stem hip implant designs: Proxima, Collo-MIS, and Minima. Postoperative bone remodeling patterns were analyzed, categorizing remodeling as bone gain, bone loss, or no observable activity, with changes tracked over time. Patient-specific biomechanical models were generated from 6-week postoperative radiographs. Finite element simulations incorporated body weight and gluteal muscle forces to estimate stress and strain distributions within the proximal femur. Strain energy was then applied to a mechanostat-based remodeling algorithm to predict bone remodeling patterns. These biomechanical predictions were compared to observed radiographic remodeling at 2 years post-surgery. A validated biomechanical model was further used to simulate different postoperative positions of the three types of stems. **Results**: No differences in bone remodeling patterns were observed among the three short-stem designs. Computational modeling demonstrated a statistically significant correlation between predicted remodeling and radiographic measurements at 2 years (*p* < 0.001). Proxima stems showed a tendency towards increased cortical bone loading under pronounced varus or valgus position in comparison to other two stems, although this observation requires further validation. **Conclusions**: This exploratory study demonstrates the feasibility of using biomechanical modeling to estimate bone remodeling around short-stem hip implants based on early postoperative radiographs. While the results are promising, they should be interpreted with caution due to the limited cohort size. The proposed modeling approach may offer clinical value in evaluating implant behavior and informing patient-specific treatment strategies. However, further research with larger populations is necessary to refine and validate these predictive tools.

## 1. Introduction

Short-stem total hip arthroplasty (THA) has become an integral component of contemporary management for hip arthritis over the past two decades. These implants have demonstrated favorable medium-term clinical outcomes and consistently low revision rates across a variety of designs [1,2,3]. With the increasing global volume of THA procedures, particularly among younger and more active patients, short-stem implants are increasingly preferred in this demographic due to their potential to preserve bone stock and soft tissue [4,5].

The heterogeneity of short-stem designs has posed challenges in developing a standardized classification system. The Joint Implant Surgery and Research Foundation (JISRF) has developed a widely accepted system based on the region of primary stability of short-stem implants. This classification categorizes stems into three primary groups: head-stabilized (Class 1), neck-stabilized (Class 2), and metaphyseal-stabilized (Class 3), with further subclassifications reflecting variations in design and surgical technique [6].

Multiple studies have reported on the capacity of short stems to achieve more natural load distribution in the proximal femur, potentially reducing stress shielding and promoting adaptive bone remodeling [7,8,9,10,11]. There, however, remains no consensus regarding the biomechanical differences between stem classes or the relative superiority of any class in facilitating physiological load-bearing.

Although prior studies have employed dual-energy X-ray absorptiometry (DEXA) to evaluate changes in bone mineral density (BMD) around short-stem implants [8,12,13], no studies to date have sought to correlate these density changes in individual patients with computational biomechanical models of load transfer using 6-week postoperative radiographs. By integrating radiographic data with finite element analyses, this study seeks to advance understanding of the relationship between implant design, load transfer, and bone remodeling in short-stem THA.

This study hypothesizes that a biomechanical model can reliably predict the progression of proximal femoral remodeling based on early postoperative radiographs. To test this hypothesis, radiographic patterns of bone remodeling across different short-stem implant classes were compared and correlated with computational biomechanical models of load transfer.

The advantage of a biomechanical model is the possibility to perform variability analysis of implant positioning in an individual patient. It is further hypothesized that the variation in implant position is a decisive parameter in determining load transfer regardless of the geometry of the implant.

## 2. Materials and Methods

### 2.1. Study Design

This study evaluated bone remodeling associated with three distinct short-stem implant designs: Proxima (DePuy Synthes), Collo-MIS, and Minima (both by LimaCorporate). These stems were chosen to represent different classes of short-stem designs according to the JISRF classification. The Proxima stem (Class 2B) is a short lateral-engaging stem with a sintered microbead surface to maximize primary and secondary fixation. Although discontinued, the Proxima stem remains a unique representative of its class. The Collo-MIS stem (Class 2A) is a short curved, neck-sparing design with a hydroxyapatite coating on its proximal two-thirds for osseointegration while minimizing distal fixation by utilizing a polished tip. The Minima stem (Class 3A) is a triple-tapered, metaphyseally stabilized design with a porous titanium surface (200 µm pore size) in the metaphyseal region to promote bone ingrowth and a roughened distal surface that allows for bone ongrowth. For all implants, ceramic heads were used, with the Pinnacle acetabular cup used in combination with the Proxima stems and the Delta PF cup used with the Collo-MIS and Minima stems.

### 2.2. Patient Selection and Follow-Up

This retrospective, single-surgeon, consecutive case series conducted at a single tertiary center was approved by the institutional ethics board. The study included patients who underwent surgery utilizing the Proxima, Minima, and Collo-MIS short stems. Overall, there were 31 implanted hips of both the Minima (29 patients) and Collo-MIS (26 patients) stems and 13 hips of the Proxima stem (9 patients). The indication for THA was primary osteoarthritis in all patients. All patients were mobile before the surgery with varying levels of sports activity.

No patients experienced complications such as joint dislocation, periprosthetic joint infection, or periprosthetic fracture. Patients with major comorbidities based on the preoperative risk assessment based on the American Society of Anesthesiologists classification were excluded from the study. Any patients with previously diagnosed osteoporosis or clear signs of osteoporosis on radiographs were also excluded from this study. Inclusion criteria required clear radiographic evidence of remodeling indicative of biomechanical strain. Only patients with standardized radiographic and clinical follow-up evaluations at 6 weeks, 6 months, 1 year, and annually thereafter were included. The cohort sizes were normalized, with five patients allocated to each group.

After surgery, all patients offloaded with crutches for 6 weeks, where after a radiological and clinical follow-up, they were allowed to bear full weight. All patients were instructed on post-surgical activity, where they were advised to avoid high impact sports activities such as running, basketball, or football. Instead, low-impact sports activities were recommended.

Radiographic evaluations focused on changes indicative of stress shielding, cortical hypertrophy, or new trabecular formation. Bone remodeling patterns were analyzed in modified Gruen zones [14] on radiographs taken at 6 weeks and 2 years postoperatively. Remodeling was categorized as bone resorption, bone apposition, or no activity. Demarcation of zones followed standardized anatomical landmarks, ensuring consistent assessment across implant designs.

### 2.3. Biomechanical Modeling

Finite element analysis (FEA) was employed to develop patient-specific planar biomechanical models from 6-week postoperative radiographs for each patient. The model differentiated between cortical bone, cancellous bone, and implant material, with material properties sourced from a study by R. Hambli [15]. To simulate physiological loading conditions relevant to everyday activities, we applied a joint contact force equivalent to 2.38 times the patient’s body weight (BW). This loading magnitude corresponds to the peak hip joint force in an average adult patient typically observed during normal walking at speed about 4 km/h [16]. The load was applied at the center of the femoral head, with the reaction force of the effective abductor muscles applied to the greater trochanter. Pauwel’s angle of the loading force [17] was adjusted for each patient to account for anatomical variability.

Strain energy density (SED) was evaluated at the interface between implant and bone and used as an input for mechanostat-based remodeling predictions (Frost’s theory) [18]. Specifically, SED divided by cancellous bone density stands for mechanical stimulus [19].

The mechanobiological model employed in this study posits the existence of upper and lower thresholds of mechanical stimulus [20]. The brute-force algorithm was used to find out the threshold value for apposition. The algorithm systematically tests all possible threshold values to find the one that maximizes classification accuracy. When the mechanical stimulus exceeds the upper threshold (12 J/kg), bone density increases through apposition. Conversely, if the stimulus falls below the lower threshold (2 J/kg) represented by the lower resorption occurrence IQR bound, bone resorption occurs. The range between these thresholds, referred to as the “lazy zone,” is characterized by negligible or minor remodeling activity. To align the biomechanical predictions with radiographic observations, each Gruen zone was categorized based on the predominant mechanical stimulus as indicative of bone apposition, bone resorption, or no activity. This approach allowed for a direct comparison between the computationally derived remodeling patterns and observed radiographic changes.

Using the mechanobiological model, we assessed how the position of the implant affects the bone remodeling in specific patients. For each of the three stems in a single patient, an implantation was numerically simulated with the stems in either varus, neutral, or valgus position. The valgus or varus position of the stem was determined by the surgeon based on radiographic positions for each stem observed in clinical practice.

### 2.4. Statistical Analysis

To evaluate the correlation between biomechanical predictions and observed remodeling patterns, a Mann–Whitney–Wilcoxon test was applied. Predicted remodeling patterns in the Gruen zones were compared to radiographic findings at 2 years post-surgery. The Chi-squared test was used for differentiation among the types of stems based on remodeling status. Statistical significance was set at *p* < 0.05.

The median value of mechanical stimulus was computed in each Gruen zone using numerical simulation data. Statistical correlation was conducted between median values of mechanical stimulus and clinical observations of three types of remodeling modes from 2-years postoperative radiographs.

## 3. Results

### 3.1. Biomechanical Model Predicts Long-Term Femoral Bone Remodeling

**Figure 1:**
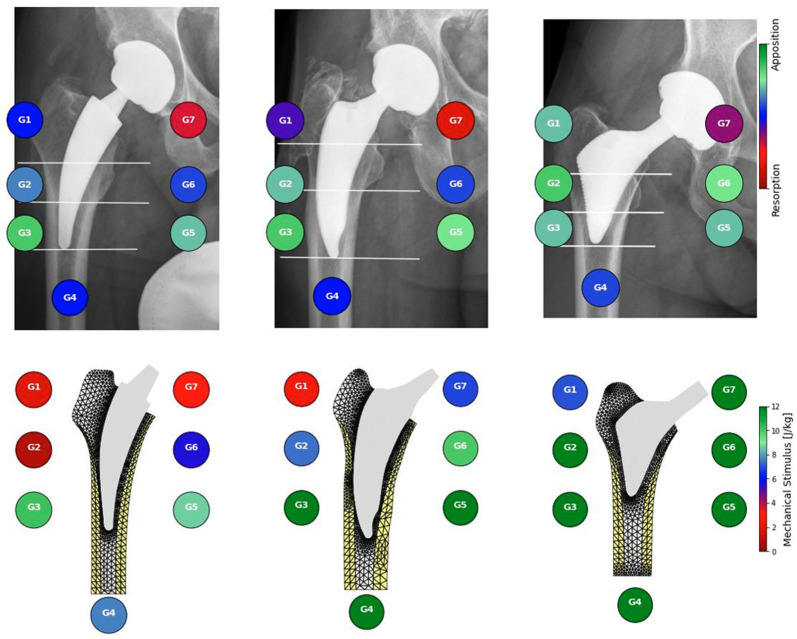
Comparison between bone activity observed in radiographs (**top**) and mechanical stimulus resulting from mechanical simulations (**bottom**) with three different short stems: Collo-MIS (**left**), Minima (**middle**), and Proxima (**right**).

**Figure 2:**
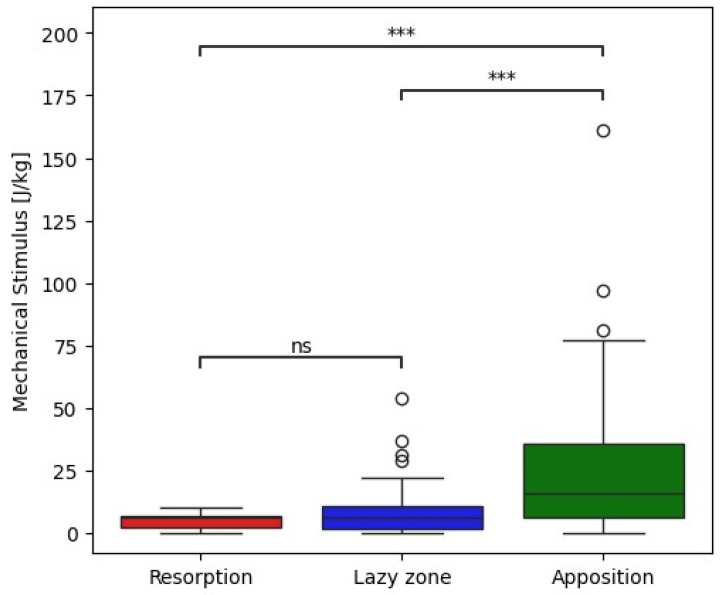
Box plot showing correlation between measured mechanical stimulus and corresponding bone remodeling changes observed in radiographs with statistical analysis. *** −1 × 10^−4^ < *p*, ns—not significant.

The mechanical stimulus at the implant–bone interface estimated from postoperative radiographs correlates with clinically observed radiographic remodeling patterns two years postoperatively (Figure 1). Mechanical stimulus was significantly higher in zones with observed apposition that in zones without observable remodeling or radiographically observed resorption (Mann–Whitney–Wilcoxon test with *p* value *p* < 0.001, Figure 2). Our preliminary findings suggest that bone apposition may be influenced by mechanical stimulus in the context of patient-specific biomechanical analysis. However, no statistically significant association was found between the overall observed remodeling patterns and the type of stem used (Chi-square test, *p* = 0.44). These results should therefore be interpreted with caution, as the limited cohort size may have constrained the ability to detect subtle differences between implant designs. Proxima stems exhibited the highest proportion of new bone apposition in zones 1 and 6 while the Minima and Collo-MIS stems showed more frequent bone loss or inactivity in these zones as also predicted by the biomechanical model (Table 1). All implants demonstrated bone resorption or inactivity in zone 7 both in clinical findings and numerical models except the Proxima stem in numerical simulations and the Col-lo-MIS stem showing the most pronounced resorption in radiological observations (Figure 1 and Table A1).

### 3.2. Stem Positioning Is Related to Bone Remodeling

**Figure 3:**
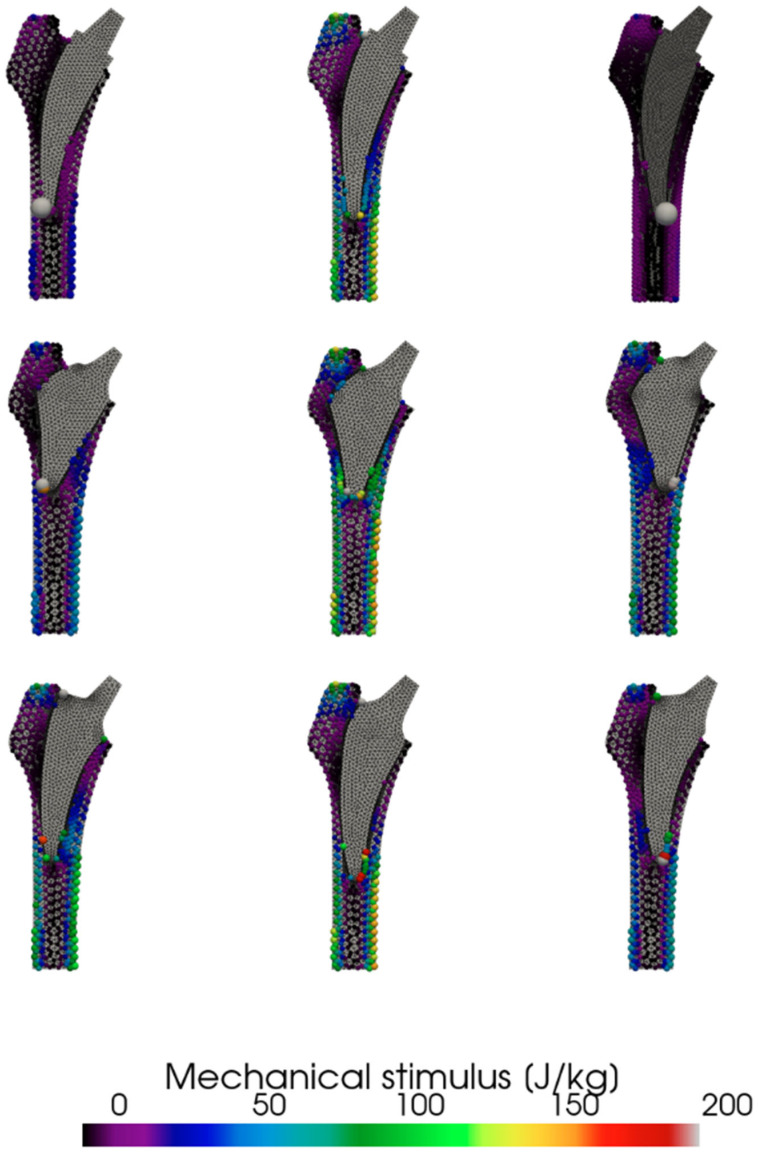
Effect of position of individual stems on observed calculated mechanical stimulus. Collo-MIS (**top**), Proxima (**middle**), Minima (**bottom**).

Simulated surgical malpositioning, in both valgus and varus alignments, resulted in cortical bone overload (Figure 3). However, the extent of this overload varied considerably among the tested stem designs. While the Collo-MIS and Minima stems demonstrated tolerance to both valgus and varus malpositioning, the Proxima stem exhibited localized cortical bone overload when malpositioned. Specifically, the biomechanical stimulus at the stem bone interface, under malaligned conditions, exceeded normal loading levels, potentially leading to bone cortical hypertrophy.

## 4. Discussion

In this study, we evaluated the bone remodeling of three different classes of short stems at two years post-surgery. While other studies focus mostly on comparison between stems of the same class, our study compares short stems of three different classes. We showed that all three stems showed very similar remodeling changes with most new bone apposition being observed in the Gruen zones G3 and G5 both in clinical results and numerical simulations. There were no statistically significant differences in remodeling between the stems. Our simulations suggest that the highest biomechanical strain may occur in zones G3 and G5 when using the Proxima stem, with the predicted bone stimulus appearing to be influenced by both stem positioning and implant classification. However, these observations should be interpreted cautiously due to the exploratory nature of the study and the limited sample size.

### 4.1. Limitations

This study included only three different types short stems. These, however, were of three separate classes with differing modes of primary fixation. This allows for their direct comparison in clinical and biomechanical performance (Figure 1). While the Proxima stem is not produced any more, we consider the knowledge of its biomechanical properties to be important for further development of new short stem designs of class 2a. All implants were implanted using free hand surgery implantation of stems without robotic assistance by one surgeon with extensive experience. Our study indicates the advantage of navigated surgery of short-stem THA to achieve accurate implant placement (Figure 3). However, the comparison in Figure 3 is based on analysis of single patient geometry with extreme varus and valgus position. The results should be therefore considered as qualitative and may differ for patients with different bone geometry or quality and/or different implant position. As the implant position was estimated from a planar radiograph, its three-dimensional antero-posterior inclination was not measured. Large anterior or posterior inclination of the implant would be exhibited by its shorter length on the radiograph [21], which was not observed in patients included in our study. It was shown in a cadaveric study that even a small deviation from the optimal position could influence primary stability [22].

The cohort size with this study was limited by the requirement of a single surgeon performing all surgeries. This potentially reduced variations due to surgeon experience or technique but considerably reduced the available number of patients. As a result, the statistical power of the study is limited. We acknowledge that with a larger patient cohort, it is likely that clearer and potentially statistically significant differences in mechanical stimulus—particularly between bone resorption and no activity, as suggested in Figure 3—could emerge. The majority of patients were excluded from this study due to missing two- year postoperative radiographs. When considering a follow-up study, it should be designed as a prospective study with rigorous follow-up procedures and periods. Such a study would also allow evaluation of time-dependent remodeling during the early and late postoperative periods.

A key limitation of this study is that the biomechanical analysis was based entirely on 2D postoperative radiographs, as no 3D imaging (e.g., CT) was available. Consequently, the finite element models were constructed and analyzed in two dimensions, and the remodeling predictions were derived from 2D strain energy distributions. While this approach simplifies the inherently 3D nature of bone remodeling, it enables consistent modeling across a broader patient population, where 3D imaging is often not part of routine clinical follow-up. CT data would allow design of three-dimensional models with location and patient-specific bone material parameters [23]. Moreover, even when CT imaging is available, its quality in the presence of metallic implants is frequently compromised due to beam-hardening artifacts and scattering, which can significantly distort bone geometry and density information. As such, 2D radiographs remain a more practical and reliable source for postoperative modeling in many clinical settings. 

We have shown that apposition level is dependent on increased mechanical stimulus, but it can also lead to pathological overload. In the remodeling estimation based on standard radiographs and a single-parameter biomechanical model, we did not differentiate between pathological overload and trabecular formation. The model could be further improved by adding an additional level of mechanical stimulus indicating bone overload that should be verified by additional DEXA measurements.

### 4.2. Biomechanical Model Predicts Long-Term Femoral Bone Remodeling

Our preliminary results suggest that the biomechanical model may be capable of estimating bone apposition based solely on a postoperative radiograph taken 6 weeks after surgery, even though the model does not explicitly incorporate biological processes such as bone metabolism or remodeling. These results are supported by independent measurement of bone density measurement by the DEXA method in another study, showing bone apposition in regions 3 and 5 [13]. The model was not able to significantly differentiate between the bone loss or the lack of activity observed from radiographs. The only zone where bone resorption was reliably observed was in zone 7. Our results support observations that short stems prevent stress shielding by design when compared to standard stems [8,13].

### 4.3. Stem Positioning Is Related to Bone Remodeling

Simulation of different stem positions revealed noticeable variations in cortical bone loading among the three designs. In particular, the Proxima stem appeared to exhibit a tendency toward increased cortical loading under extreme varus or valgus alignment. However, given the exploratory nature of the study, these findings should be interpreted with caution and warrant further investigation in larger cohorts. We assume that this observation is due to the short design of the implant, leading to a sharp point contact of the implant and cortical bone, which leads to such high measured strain values. This correlates with clinical observations where cortical hypertrophy was observed in clinical cases with these stem positions [5]. For clinical application, this implies that the Proxima stem is susceptible to these pathologic remodeling modes when implanted in abnormal positions. Some studies have pointed out the difficulty in proper stem alignment with the Proxima stem and recommend its use only by experienced surgeons [24].

In the Collo-MIS stem, we observed much smaller stress values in general (Figure 2). We understand this to mean that the load is distributed more efficiently through the stem to the endosteal bone of the metaphysis. The Collo-MIS stem as part of its design utilizes the neck as part of its primary fixation while using impactors to compress the cancellous bone into the shape of the implant. This means that the stem should not be in direct contact with cortical bone distally but has a layer of compressed cancellous bone allowing for remodeling into trabecular structures that may transfer load in a more physiological way. In a clinical study of Collo-MIS stems, CCD angle measurements showed a mean +2° variation compared to mean physiological values in 60% of cases, and no cortical overloading was observed [25]. These results corroborate with our numerical results showing no cortical overload in the neutral position (Figure 3).

The Minima stem (class 3a) shows no considerable differences between the varus, valgus, and neutral positions with the maximum strain being transferred to the lateral cortex in G3 Gruen zone in all positions. We assume this is due to the shape of the stem and method of implantation. The shape allows for deep implantation of the stem and wide cortical contact in all positions. Clinically, this means that the Minima stem potentially tolerates implantation in abnormal positions and can be more suited to general use even with less experienced surgeons. This method of primary fixation, however, requires direct contact between the stem and distal cortical bone, which can lead to bone overloading clinically presenting as cortical hypertrophy [26].

On the radiographic observations, there were several different modes of bone formation with some showing formation of new trabecular structure attaching the surface of the stem if there was not in contact with cortical bone which allowed for formation of trabecular structures. In other situations, cortical hypertrophy was observed if either the medial or lateral surfaces of the stem were in direct contact with cortical bone. It can be assumed that trabecular bone is better adapted to transfer of force through it when compared to cortical bone, explaining this difference in new bone formation.

These preliminary findings suggest that bone remodeling may be more strongly influenced by stem positioning than by stem type alone. Certain stem designs appear to be more sensitive to malpositioning, which could lead to less favorable remodeling outcomes. In our simulations, the Proxima stem showed a greater dependency on accurate alignment to avoid such effects, whereas the Minima stem appeared more tolerant to positional variation. However, these trends require further validation in larger, clinically diverse cohorts.

## 5. Conclusions

Our results indicate that the biomechanical model may be capable of predicting bone remodeling patterns in the proximal femoral cortex following implantation of various short-stem THA designs. While the Proxima stem showed favorable trends in both biomechanical response and clinical indicators within our limited cohort, these findings should be interpreted as preliminary and require further validation in larger, more diverse patient populations. The applicability of the model was demonstrated in the analysis of three types of short-stem implants with three types of position, namely neutral, varus, and valgus in a single patient. This lays down a foundation for improving patient-specific preoperative planning by using patient radiographs to predict bone remodeling over time, based on stem position. Our findings suggest that implant positioning may play a more critical role in biomechanical outcomes than implant type alone. For example, the Collo-MIS stem demonstrated favorable biomechanical behavior in neutral alignment but showed increased cortical loading under extreme varus or valgus positioning compared to the other designs. These observations highlight the potential importance of precise surgical technique—such as the use of centering impactors or navigation-assisted implantation—particularly when using the Collo-MIS stem. In contrast, the Proxima and Minima stems appeared more tolerant to positional variation in our simulations. This study indicates that the combination of clinical data and patient-specific models has potential in predicting implant performance and optimizing patient-specific treatment and follow-up strategies. Further research is warranted to refine and validate these predictive tools in larger patient cohorts.

## Figures and Tables

**Table 1:** Patient data set description.

Gender (M:F).	Height (cm)	Weight (kg)	BMI	Age at Surgery
4:11	168 (IQR: 158.25–176.25)	70 (IQR: 47.5–99.5)	25.9 (IQR: 14.75–37.15)	62 (IQR: 41.25–79.25)

## Data Availability

Dataset available on request from the authors.

## Abbreviations

The following abbreviations are used in this manuscript:

FEA	Finite Element Analysis
SED	Strain Energy Density
CT	Computed Tomography
THA	Total Hip Arthroplasty
JISRF	Joint Implant Surgery and Research Foundation
DEXA	Dual-Energy X-ray Absorptiometry

## Appendix A

**Table A1:** Radiographic observations.

Zone	Stem Type								
	Collo-MIS			Minima			Proxima		
	Resorption	Lazy Zone	Apposition	Resorption	Lazy Zone	Apposition	Resorption	Lazy Zone	Apposition
G1	20%	60%	20%	20%	80%	Not observed	Not observed	40%	60%
G2	20%	20%	60%	Not observed	40%	60%	Not observed	Not observed	100%
G3	Not observed	Not observed	100%	Not observed	Not observed	100%	Not observed	40%	60%
G4	Not observed	100%	Not observed	Not observed	100%	Not observed	Not observed	80%	20%
G5	Not observed	40%	60%	Not observed	20%	80%	Not observed	40%	60%
G6	20%	40%	40%	Not observed	80%	20%	Not observed	20%	80%
G7	60%	40%	Not observed	100%	Not observed	Not observed	40%	60%	Not observed
